# Effect of fermented rapeseed meal in the mixture for growing pigs on the gastrointestinal tract, antioxidant status, and immune response

**DOI:** 10.1038/s41598-022-20227-2

**Published:** 2022-09-21

**Authors:** Anna Czech, Bożena Nowakowicz-Debek, Marcin Łukaszewicz, Mariusz Florek, Mateusz Ossowski, Łukasz Wlazło

**Affiliations:** 1 Department of Biochemistry and Toxicology, Faculty of Animal Sciences and Bioeconomy, University of Life Sciences in Lublin, Akademicka 13, 20-950 Lublin, Poland; 2 Department of Animal Hygiene and Environmental Hazards, Faculty of Animal Sciences and Bioeconomy, University of Life Sciences in Lublin, Akademicka 13, 20-950 Lublin, Poland; 3 Department of Biotransformation, Faculty of Biotechnology, University of Wroclaw, F. Joliot-Curie 14A, 50-383 Wrocław, Poland; 4 Department of Quality Assessment and Processing of Animal Products, Faculty of Animal Sciences and Bioeconomy, University of Life Sciences in Lublin, Akademicka 13, 20-950 Lublin, Poland

**Keywords:** Biological techniques, Immunology, Microbiology, Physiology

## Abstract

The ban on the use of zinc oxide has increased interest in probiotics, prebiotics, synbiotics and organic acids, as well as fermented components in the diet of weaned piglets. This study assessed the effect of 8% fermented rapeseed meal in weaner diets on characteristics of the gastrointestinal tract, the small intestinal microbiota, and immune and antioxidant status. The effects were determined by measuring biochemical and haematological blood parameters, levels of class G, A and M immunoglobulins and IL-6, and the antioxidant potential of the plasma. After slaughter, the gastrointestinal tract was measured, the viscosity of the digesta was determined, and microbiological tests were performed. The results showed that the fermented component reduced the viscosity of the digesta and the length of segments of the gastrointestinal tract. It caused a statistically significant increase in lactic acid bacteria and a decrease in total bacteria. The haematological and biochemical analyses of the blood confirmed the biological activity of the fermented component. Pigs from group FR had significantly higher haemoglobin levels (p = 0.001), RBC count (p = 0.015), and haematocrit (Ht) value (p < 0.001) than the control animals. A diet including 8% rapeseed meal fermented using *Bacillus subtilis* strain 87Y benefits gastrointestinal function by stabilizing and improving the function of the bacterial microbiota, inhibiting growth of certain pathogens, and strengthening immunity.

## Introduction

In modern pig farming systems, suitable rearing conditions and a balanced diet that meets the animals’ nutritional needs at each stage of growth are essential to achieving their full genetic potential. Particular attention is focused on the post-weaning period in pigs, when they are subjected to numerous stressors. This is an important period in pig rearing, as it is often accompanied by temporary low feed intake, poor growth, intestinal dysbiosis, and post-weaning diarrhoea (PWD), which pose a threat to the animals’ health and welfare, increase mortality, and lead to economic losses^1–3^. In addition, exposure to stressors in conditions of intensive production can disturb the redox balance and induce the generation of reactive oxygen species (ROS). This leads to oxidative stress, which can result in the development of various diseases^4^. For this reason the ingredients and additives used to prepare the post-weaning feed are extremely important. They must be easily digestible, maintain the lowest possible pH in the gastrointestinal tract, and also prevent the multiplication of pathogenic *Escherichia coli* bacteria, which cause diarrhoea in piglets^3^. For many years the problem of PWD was addressed by using zinc oxide in the diet of weaned piglets. Large amounts of zinc oxide added to the feed (2500 to 3000 mg Zn/kg diet) were used to prevent PWD^5^. However, the adverse effect of long-term elevation of the Zn^2+^ content in the faeces and urine of pigs poses a potential threat to the environment^6^. There are reports that zinc oxide in animal production can be successfully replaced with probiotics, prebiotics, synbiotics, or organic acids^6,7^. Owing to their interesting chemical composition, interest in the use of fermented components in the diet of post-weaning piglets has also recently been growing^8–10^. Studies have been conducted mainly on fermented soybean meal (FSBM)^11–14^. An interesting partial replacement for FSBM may be fermented rapeseed meal (FRSM), which has the added value of not being obtained from GMO crops.

Fermentation is a process which improves the quality of rapeseed meal (RSM). It increases the bioavailability of protein, promotes the synthesis of vitamins and antioxidants, and reduces the content of harmful compounds such as glucosinolates, phytates and non-starch polysaccharides^10,15–18^. According to Rozal et al.^19^ fermentation causes the degradation of 84% of carbohydrates, 30% of lignin and 47% of all glucosinolates in RSM. Owing to the presence of probiotics (lactobacilli or bifidobacteria), prebiotics (lactic acid), or organic acids, RSM has multi-faceted effects on the animal’s body. A few studies show that FRSM has antimicrobial effects (inhibiting growth or killing the cells of various microorganisms)^20,21^, and immunoregulatory effects, enhancing or suppressing the immune system according to need^22,23^. It also neutralizes bacterial toxins, regulates redox processes by increasing or inhibiting the formation of ROS, and regulates metabolism of minerals, including their absorption from food^24^. Previous studies have shown that increased concentrations of biologically active compounds, including antioxidants, in FRSM^15^ can have many benefits in the rearing of poultry^18^, rabbits^25^, and fish^20,23^.

Fermented feed has a low pH and contains high concentrations of lactic acid, several volatile fatty acids (VFA), e.g. acetic, butyric and propionic acid, and a large number of lactic acid bacteria. These four parameters, independently or in combination, can affect the bacterial composition of the gastrointestinal tract. Undissociated forms of lactic acid and VFA are believed to play a role in reducing the number of *Enterobacteriaceae*, including *Salmonella* spp. The reduction in *Salmonella* may also be due to the lactobacilli supplied with the feed, as they compete with potentially pathogenic bacteria in the gut by enhancing resistance to colonization^26^.

To increase the nutritional value of FRSM, many microbial species, such as *Aspergillus oryzae*^27^, lactic acid bacteria^15^ and *Bacillus subtilis*^28^, have been used independently for the fermentation process^11^. Experiments in animals do not clearly indicate which microorganisms are most effective^29^. Our own research on mink^30^, rabbits^25^ and poultry^21^ indicate that the *B. subtilis* 87Y strain used for FRSM both enriches the product and helps to maintain homeostasis and prevent dysbiosis, thus improving production outcomes.

In the present study we postulated that an 8% addition of RSM fermented using *B. subtilis* strain 87Y in the diet of weaners, owing to its antimicrobial, immunoregulatory and antioxidant properties, can prevent PWD and thus serve as a replacement for zinc oxide.

Therefore, the aim of the study was to analyse the effect of 8% FRSM in the diet of weaners on characteristics of the gastrointestinal tract, the microbiota of the small intestine, and immune and antioxidant status.

## Results

The viscosity of the intestinal contents was lowest in the pigs from group FRA (weaners receiving a diet with 8% FRSMb and feed additives) vs groups C and FR (weaners receiving a diet with 8% FRSMb without feed additives) (p = 0.002). The stomach, small intestine and large intestine of the animals from the FRA treatment were significantly longer than in the control treatment. The stomachand large intestine of the animals from group FR were significantly longer than in the control group (Table 1). The lengths of the stomach and large intestine were similar in animals from groups FRA and FR and significantly higher than in group C. This also applied to the total length of the gastrointestinal tract (p = 0.001). The small intestine was longest in group FRA vs groups C and FR (p = 0.037). The pH of all sections of the gastrointestinal tract (except the large intestine and duodenum) was significantly lower in group FRA than in the control. In the weaners from group FR, the pH of the contents of the middle part of the small and large intestine differed statistically significantly from group C (Table 1).

**Table 1:** Characteristics of the gastrointestinal tract (GIT) of weaners.

Parameter	C	FRA	FR	SEM	P_value_
**Length of GIT sections**					
Stomach	14.36^b^	18.75^a^	17.33^a^	0.531	< 0.001
Small intestine	14.25^b^	16.45^a^	15.14^ab^	0.372	0.043
Large intestine	1.80^b^	2.19^a^	2.23^a^	0.077	0.030
Total	30.41^b^	37.39^a^	34.70^a^	0.895	0.001
Dynamic viscosity (Pa s) temp 37–38	1.25^b^	0.187^a^	1.07^b^	0.149	0.002
Shear rate (1/s)	1.36	1.01	1.33	0.102	0.312
**pH of GIT sections**					
Stomach	4.34^a^	3.60^b^	4.18^a^	0.094	< 0.001
Beginning of small intestine	6.33^a^	5.79^b^	6.39^a^	0.081	< 0.001
Middle of small intestine	6.76^a^	6.04^b^	6.15^b^	0.089	< 0.001
End of small intestine	6.60^a^	5.82^b^	6.48^a^	0.093	< 0.001
Beginning of large intestine	6.13	5.67	5.95	0.060	0.061
Middle of large intestine	5.69^b^	5.70^b^	6.11^a^	0.072	0.014
End of large intestine	5.90	5.61	5.97	0.049	0.002

Feeding groups: *C* control group, *FRA* weaners receiving a diet with 8% FRSMb and feed additives, *FR* weaners receiving a diet with 8% FRSMb without feed additives.

^a,b^Means within a row with different superscripts differ significantly (p ≤ 0.05).

The total number of aerobic mesophilic bacteria in the gastrointestinal contents was lowest in the animals from group FRA in comparison to the control and FR treatments (p = 0.027). In groups FRA and FR there was also a statistically significant increase in the total number of lactic acid bacteria of the genus *Lactobacillus* compared to group C (p = 0.006). No statistically significant differences between groups were noted for the remaining parameters of the microbiological composition of the digesta. The analysis did not reveal the presence of *Salmonella* bacteria in any of the material (Table 2).

**Table 2:** Microbiological composition of the contents of the small intestine of weaners.

Parameter	C	FRA	FR	SEM	P_value_
Total aerobic bacteria	7.3 × 10^4a^	1.2 × 10^6c^	1.6 × 10^5b^	198,225.8	0.027
Total yeasts and moulds	1.3 × 10^3^	6.3 × 10^3^	8.5 × 10^3^	1564.5	0.258
Total *Clostridium perfringens*	1.9 × 10^3^	1.2 × 10^3^	1.5 × 10^3^	286.97	0.698
Total *Lactobacillus*	5.7 × 10^6b^	1.6 × 10^7a^	3.2 × 10^7a^	3,559,096	0.006
Coliform bacteria	4.6 × 10^4^	3.6 × 10^4^	4.4 × 10^4^	6249.6	0.796
*Escherichia coli*	4.5 × 10^4^	3.3 × 10^4^	4.1 × 10^4^	6370.0	0.431
*Salmonella*	ng	ng	ng	–	–

Feeding groups: *C* control group, *FRA* weaners receiving a diet with 8% FRSMb and feed additives, *FR* weaners receiving a diet with 8% FRSMb without feed additives.

*SEM* standard error of the mean, *ng* no growth.

^a,b,c^Means within a row with different superscripts differ significantly (p ≤ 0.05).

Pigs from group FR had significantly higher haemoglobin levels (p = 0.001), RBC count (p = 0.015), and haematocrit (Ht) value (p < 0.001) than the control animals. The Ht value in the pigs from group FRA was similar to that of the FR animals and significantly higher than in the control treatment (Table 3).

**Table 3:** Red and white blood cell parameters and content of immunoglobulins in the blood of weaners.

Parameter	C	FRA	FR	SEM	P_value_
**Red blood cell parameters**					
Ht; l l^−1^	0.308^b^	0.342^a^	0.347^a^	0.098	< 0.001
Hb; mmol l^−1^	5.88^b^	6.12^ab^	6.51^a^	0.081	0.001
RBC; 10^12^ l^−1^	6.38^b^	6.58^ab^	7.01^a^	0.005	0.015
**White blood cell parameters**					
WBC; 10^9^ l^−1^	25.47^a^	17.85^b^	20.66^ab^	0.830	0.046
**Leukogram**					
GRA; %	41.80^a^	25.34^b^	23.44^b^	2.11	< 0.001
LYM; %	49.64^b^	64.12^a^	67.45^a^	2.01	< 0.001
MONO; %	6.39^a^	6.54^a^	5.22^b^	0.186	0.001
EOS; %	1.06	2.85	2.85	0.241	0.088
BAS; %	1.11	1.15	1.05	0.030	0.392
GRA/LYM	0.848^a^	0.398^b^	0.350^b^	0.058	0.000
**Immunological parameters**					
IgG; mg ml^−1^	17.90^b^	25.55^a^	17.50^b^	0.959	< 0.001
IgA; mg ml^−1^	3.34^b^	5.87^a^	3.42^b^	0.295	0.044
IgM; mg ml^−1^	0.708^b^	1.02^a^	0.304^c^	0.086	< 0.001
IL6; pg ml^−1^	116.2^a^	105.1^b^	117.2^a^	6.66	0.101

Feeding groups: *C* control group, *FRA* weaners receiving a diet with 8% FRSMb and feed additives, *FR* weaners receiving a diet with 8% FRSMb without feed additives, *Hb* haemoglobin concentration, *Ht* haematocrit, *RBC* red blood cell count, *WBC* white blood cell count, *GRA* neutrophil granulocytes, *LYM* lymphocytes, *MONO* monocytes, *EOS* eosinophils, *BAS* basophils, *IgA* immunoglobulin A, *IgG* immunoglobulin G, *IgM* immunoglobulin M, *IL-6* interleukin 6.

*SEM* standard error of the mean.

^a,b,c^Means within a row with different superscripts differ significantly (p ≤ 0.05).

The levels of UA, FRAP, and vitamin C as well as CAT activity in the plasma of pigs from FRA group were significantly higher than in the control treatment. The blood of these animals had significantly lower MDA levels compared to group FR and C (p < 0.001). The animals from group FR had significantly higher levels of LOOH and MDA (p < 0.001) and significantly higher levels of CREAT, and FRAP than group C (Table 4). CAT activity were significantly higher, and the MDA level was lower in the liver of pigs from group FRA than in the control group (Table 4). The plasma concentrations of iron, copper, zinc and selenium were significantly higher (p < 0.001) in the pigs in group FRA than in the control group (C) (Table 4).

**Table 4:** Selected biochemical and redox parameters in the plasma and liver of weaners.

Parameter	C	FRA	FR	SEM	P_value_
**Plasma**					
UA; µmol l^−1^	0.333^b^	0.377^a^	0.324^b^	0.007	< 0.001
UREA; mmol l^–1^	2.45	2.49	2.92	0.060	0.130
CREAT; µmol l^−1^	86.81^b^	79.41^b^	96.55^a^	2.10	< 0.001
BIL; µmol l^−1^	2.52^a^	2.72^a^	1.83^b^	0.098	< 0.001
Iron; µmol l^−1^	19.43^b^	29.31^a^	17.86^b^	0.837	< 0.001
Copper; µmol l^−1^	30.38^c^	40.97^a^	34.58^b^	0.726	< 0.001
Zinc; µmol l^−1^	43.88^b^	53.14^a^	42.26^b^	0.983	< 0.001
Selenium; µmol l^−1^	1.11^b^	1.60^a^	1.36^ab^	0.050	< 0.001
CAT; U ml^−1^	11.28^c^	43.18^a^	28.69^b^	3.23	< 0.001
SOD; U ml^−1^	32.41	30.50	32.42	0.587	0.325
FRAP; µmol l^−1^	13.10^c^	22.15^a^	16.81^b^	0.971	< 0.001
Vitamin C, µmol l^−1^	20.31^b^	25.76^a^	23.89^ab^	1.32	0.035
LOOH; µmol l^−1^	1.97^b^	2.13^ab^	2.40^a^	0.049	< 0.001
MDA; µmol l^−1^	0.761^b^	0.332^c^	2.01^a^	0.178	< 0.001
**Liver**					
CAT; U g^−1^	162.8^b^	261.36^a^	169.2^b^	15.18	0.001
SOD; U g^−1^	90.69^b^	133.3^a^	102.4^ab^	7.08	0.019
LOOH; nmol g^−1^	12.07	10.65	9.54	0.514	0.127
MDA; nmol g^−1^	15.07^a^	10.91^b^	10.97^b^	0.737	0.011

Feeding groups: *C* control group, *FRA* weaners receiving a diet with 8% FRSMb and feed additives, *FR* weaners receiving a diet with 8% FRSMb without feed additives, *UA* uric acid, *UREA* urea, *CREAT* creatinine, *BIL* bilirubin, *SOD* superoxide dismutase, *CAT* catalase, *FRAP* total antioxidant potential, *MDA* malondialdehyde, *LOOH* peroxides.

*SEM* standard error of the mean.

^a,b,c^Means within a row with different superscripts differ significantly (p ≤ 0.05).

## Discussion

Weaning on commercial pig farms takes place suddenly, usually between 3 and 4 weeks of age. At this time the piglets are exposed to various stressors, including separation from the sow and litter, new housing conditions, and a sudden change in diet^1–3^. The consequence may be a refusal to eat, followed by too much feed intake at once^31^. This leads to an imbalance of the intestinal microbiota, which can cause diarrhoea^9^. Another cause of diarrhoea in young pigs is a poorly balanced post-weaning diet consisting of hard-to-digest ingredients, rich in anti-nutrients and containing too much crude protein^1^. High hopes for optimizing diets for piglets have recently been placed in fermented components, including FRSM. The effect of fermented components depends not only on the species of microbes used for the fermentation process but also the strain^13,32,33^.

Rapeseed meal fermented using *B. subtilis* strain 87Y not only has high nutritional value^25^ and low content of anti-nutrient substances, but also contains enzymes that improve digestion and numerous substances stimulating the development of beneficial intestinal microbes, i.e. the above-mentioned probiotics and prebiotics^34^. In the present study, FRSMb in the diet of young pigs significantly reduces the pH of most parts of the gastrointestinal tract, which was particularly evident in the pigs from FRA group. The significantly lower pH of the gastrointestinal contents in group FRA corresponded to their significantly lower viscosity, which was not observed in the FR group. This was probably due to the additional use of organic acids in group FRA Acidification of the gastrointestinal contents to pH 4.0 increases secretion of digestive enzymes and allows pepsinogen to be converted to pepsin, an enzyme that digests protein. In addition, bacteria and allergens are degraded by hydrochloric acid secreted in the stomach and pancreatic juice^35^. Besides organic acids, equally popular additives in feed for piglets are prebiotics and probiotics, which also effectively reduce the pH of the intestinal contents^7^. This is particularly important in the case of weaned piglets, as the pH of the gastrointestinal contents (the first line of defence against bacterial infection) is often too high, which can lead to poorer digestion of feed components and to the development of harmful intestinal microbes^24^.

The presence of enzymatic and pro- and pre-biotic additives and organic acids provided with FRSM (group FRA) promotes the growth of beneficial intestinal microbes, which was not observed in group FR. In the intestinal contents of the pigs from group FRA there was a significant increase in aerobic mesophilic bacteria and in the total number of lactic acid bacteria of the genus *Lactobacillus*, which was consistent with reports^22^. Studies also show that the number of LAB and yeasts and the concentrations of lactic acid and acetic acid in the intestinal contents significantly increases in pigs fed fermented feed^26,36,37^. An increase in the number of LAB when fermented ingredients were used in the diet was also noted in the gastrointestinal contents of mink^30^, rabbits^25^, broiler chickens, turkeys, and ducks^23^. Scholten et al.^36^ reported a slight increase in the number of LAB in the small intestine of pigs fed fermented liquid feed.

Studies by Ravindran and Kornegay^38^ and van Winsen et al.^26^ indicate that fermented components in feed, by reducing the pH in the entire gastrointestinal tract, increase the action of VFA on *Enterobacteriaceae* and probably on *Salmonella* spp. This was not confirmed in our experiment. While a slight increase in the total number of *Clostridium perfringens* and *Escherichia coli* bacteria was observed in the animals from groups FRA and FR, the results were not confirmed statistically. However, it can be assumed that the presence of fermented products creates an inhospitable environment for the development of pathogenic bacteria^39^. A mechanism proposed to explain the reduction in pathogens in the gut is an increase in nutrient digestibility in the small intestine due to the presence of the fermented component, resulting in fewer substrates for microbial growth in the lower gastrointestinal tract^26^. Dietary fiber is resistant to digestion by endogenous enzymes in the pig's small intestine, but may be partially or completely fermented by the microbiota in the large intestine to VFA, also likely that fermentation partially destroys the fiber content of RSM, increasing its solubility and decreasing viscosity. Increased fiber solubility makes fibers more fermentable, decreasing the pH^40^. Probiotics present in fermented products limit the development of pathogenic microbes by producing bacteriocins, hydrogen peroxide or organic acids, while at the same time reducing the occurrence of diarrhoea caused by rotaviruses, antibiotics, or *Clostridium difficile* infection^28,41^. Reducing symptoms of diarrhoea is of enormous importance in pig production, as it renders the animals less susceptible to *Escherichia coli* infection^42^. Research has shown a reduction in the number of *Enterobacteriaceae* in the entire gastrointestinal tract of pigs receiving fermented liquid feed (FLF)^26,43^. Our previous research on mink^30^, rabbits^25^ and poultry^21^ has also shown a reduction in pathogens in animals receiving FRSMb.

Fermented rapeseed meal in the diet, by reducing the pH and viscosity of the digesta and improving its microbial composition, can also cause an increase in the length of the digestive tract. All parts of the gastrointestinal tract responsible for digestion and absorption of nutrients, i.e. the stomach, and small intestine, were significantly longer in FRA and FR groups than in the control group. Studies have shown a link between diet and increased size of digestive organs, including the length of the intestines^1,44^. An increase in the surface area of the gastrointestinal tract (intestinal strands mainly in the small intestine) together with a reduction in the pH of its contents can increase the secretion of digestive enzymes. This improves digestion and absorption of nutrients, including minerals, and also stimulates the immune system^24^.

Based on the results of our previous research, we postulated that the fermented component (FRSMb), as a factor including the absorption of minerals, including zinc^34^, and stimulating immune processes^24,45^, would be effective enough that the addition of zinc to the diet of piglets could be minimized or even eliminated (group FR). This assumption was very bold, but the results enabled some interesting observations that should be further studied.

Zinc in the form of zinc oxide is included in the diet of weaned piglets to prevent PWD^5,6^. However, as the risk of contamination of the environment outweighs the benefits of the use of ZnO, the European Commission determined that it should not be used in amounts greater than 150 ppm^46^. The results of our study indicate that the amount of zinc in compound feeds resulting from its content in the feed ingredients alone (group FR) did not disturb homeostasis in the pigs. The red and white blood cell parameters in all experimental groups were within reference ranges^47^. Red blood cell parameters, i.e. Ht, Hb and RBC, as well as the lymphocyte count in the blood of the animals in group FR were even significantly higher than in the control group. The presence of enzymes (phytase, NSP enzymes), pro- and prebiotics, and organic acids in FRSMb creates an optimal environment (reduced digesta viscosity and pH) stimulating absorption of minerals, including zinc^24^. In our study, the level of zinc in the plasma of pigs from group FR was comparable to its level in the control group. In the FR piglets, however, as in the control group, there was a significant increase in the plasma level of IL6, as well as in the level of lipid peroxidation products, i.e. LOOH and MDA. The increase in IL6 and in LOOH and MDA may indicate an ongoing infection^48^. Factors stimulating the production of IL6 include pro-inflammatory cytokines, particularly interleukin 1. IL6 has a direct, strong stimulatory effect on inflammatory processes^49^.

The use of additives (group FRA) had no effect on red blood cell parameters in the piglets. This is surprising, as a significant increase in these parameters was observed in group FR. It should be noted, however, that all haematological parameters were within reference limits^47^. On the other hand, significant immune stimulation was observed in group FRA, manifested as a significant increase in the plasma level of IgG. The increase in IgG was consistent with results reported by Zhu et al.^13^. The serum IgG concentration reflects the animal’s systemic immune status^50^. In our study, the lymphocyte count and IgG, IgA and IgM levels were increased as well, by 29.2%, 42.7%, 75.7% and 44.1%, respectively, vs the control group. Lymphocyte proliferation is an important stage of the immune response, and the proliferative response is antigen-specific^14^. Stimulation of immune processes accompanied by an increase in the lymphocyte count and the levels of class A and G immunoglobulins has also been observed in pregnant and lactating sows and piglets whose diet contained fermented components^8,51^. According to Feng et al.^52^ and Tang et al.^53^, fermentation can increase levels of small peptides in fermented RSM and improve animals’ immune status. Previous studies^54^ have shown that fermented protein feedstuffs are a rich source of polysaccharides, bioactive compounds, and lactic acid bacteria, which promote immunoglobulin production and inhibit the release of pro-inflammatory cytokines in animals. In the present study, the FRA diet significantly raised the IgM level in the blood. IgM is the first antibody isotype produced in the primary antibody response and is the main class of immunoglobulins expressed on the surface of lymphocytes^55^. IgM can be used as an indicator of exposure to pathogens^56^. Research shows that IgM in the plasma can fall or rise in response to immunosuppression and immunostimulation. In most cases, an elevated IgM level is observed during infection, accompanied by elevated levels of IgG and the pro-inflammatory cytokine IL-6, and oxidation reactions take place in the cells^55,57^. In our study, the FRA diet did not increase the level of IL-6 or indicators of oxidative stress. Therefore the increase in IgM may only indicate stimulation of the immune system.

The increase in the Cu, Zn and Se levels in the blood of piglets from FRA group indicates their beneficial effect on antioxidant status. CuZn-SOD (superoxide dismutase), which is found in the cytosol, contains about 0.30% Cu and 0.25% Zn and plays a key role in defending cells from the harmful effects of peroxides^58^. In our study, SOD activity in the liver increased in the FRA group. The other parameters, including the concentrations of low-molecular-weight antioxidants, i.e. UA and vitamin C, as well as BIL, CAT activity, the FRAP value, and especially the concentrations of LOOH and MDA in the plasma, also indicate that FRSMb (group FRA) has a positive effect on the antioxidant status of piglets.

MDA is a lipid peroxidation end product, and thus its level can be used to monitor the degree of lipid peroxidation by ROS^59^. The decrease in the MDA concentration in our experiment was linked to a decrease in the level of lipid peroxidation end products. Pro- and prebiotic additives may have a positive effect on the markers of oxidative stress, including the reduction of MDA concentrations^60^.

The beneficial effects of FRSM may be linked to the chemical composition of RSM, which contains phenolic acids with strong antioxidant properties^15^. According to He et al.^15^, the antioxidant properties of phenolic acids in RSM inhibit oxidation, thereby maintaining its high nutritional quality. The increase in antioxidant activity in the pigs receiving FRSMb may also have been due to improved protein breakdown, especially following digestion by trypsin, as shown by and Yoshie-Stark et al.^61^.

The literature contains few studies assessing the effect of FRSM on the redox status of piglets. In a study by Hu et al.^62^, SOD activity and total antioxidant capacity were significantly higher in the blood of broiler chickens fed fermented RSM in comparison to non-fermented RSM, but no significant differences were noted for other parameters of redox status. Czech et al.^51^ observed a decrease in MDA and LOOH levels accompanied by an increase in CAT activity in lactating sows fed a diet containing a component fermented using Lactobacillus bacteria, as well as an increase in the antioxidant potential of the plasma (FRAP). Wang et al.^63^ tested the effect of a probiotic strain of L. fermentum on pigs. They showed that supplementation with this microorganism promotes healthy growth in pigs by increasing SOD activity and reducing MDA concentrations in the serum. Moreover, they observed an increase in CAT activity and a decrease in the MDA concentration in the liver of these animals.

## Conclusions

The use of a diet with 8% RSM fermented using *B. subtilis* strain 87Y together with enzymatic, probiotic and prebiotic additives, organic acids, and 150 ppm zinc has beneficial effects on the functioning of the gastrointestinal tract.

It helps to stabilize the sensitive intestinal mucosa, influences the diversity and function of the bacterial microbiota and strengthens the animals’ immune response.

The inclusion of FRSMb in the diet together with feed additives and 150 ppm ZnO may determine immune response enhancing immunoglobulin synthesis in the piglet.

The use of FRSMb in the diet of piglets without feed additives or 150 ppm ZnO does not improve immune parameters, but also does not disturb them. This is a very valuable observation, especially at a time when medicated feeds are going out of use. At this stage of research, however, it cannot be conclusively stated whether FRSMb can entirely replace pro- and prebiotics, organic acids, and enzyme additives in the diet of piglets, in part because the increase in the levels of IL-6 and of LOOH and MDA may be indicative of infection.

## Methods

The study was carried out in compliance with the ARRIVE guidelines.

All methods were carried out in accordance with relevant guidelines and regulations regarding to life animal studies and the procedures complied with the Directive 2007/526/EC of the European Parliament and of the Council on the protection of animals used for scientific purposes.

All tests on animals were performed with the approval of the Local Ethics Committee on Animal Experimentation of the University of Life Sciences in Lublin, Poland (approval no. 50/2018 of 1 April, 2018).

### Pigs, management, and diets

A total of 288 Yorkshire × Danish Landrace crossbred pigs (144 barrows and 144 gilts) were randomly assigned to three diet treatments, with 6 replicates each and 16 pigs (8 barrows and 8 gilts) in each replicate.

The experiment was begun when the pigs were 28 days old (after weaning) and completed when they reached a body weight of about 35 kg. Before the experiment, the pigs were individually tagged and weighed. Variation in body weight within and between each pen and group replicate was minimized as far as possible.

In the control group (C), soybean meal (SBM) was the main source of dietary protein, while the remaining groups (FRA and FR) received diets in which SBM was partially replaced with 8% RSM fermented with *B. subtilis* strain 87Y (FRSMb). The procedure for fermenting RSM is described in Wlazlo et al.^25^. The FR diet, in contrast to the FRA treatment, did not contain enzyme additives, pro- and prebiotic additives, organic acids, or added zinc (Table 5).

**Table 5:** Ingredient composition (% of air-dry matter) and chemical composition of piglet.

Ingredient^1^	C	FRA	FR
Wheat	60.48	68.33	58.8
Barley	20	20	20
Soybean meal, 46.5% protein	9.24	3.46	3.32
Fermented rapeseed meal-FRSMb	0	8	8
Fish meal 65%	4	4	4
Soybean oil	2.1	2.13	2.13
Chalk	0.95	0.87	0.87
L-Lysin∙HCl, 78%	0.82	0.91	0.91
L-Threonine	0.37	0.4	0.4
DL-Methionine	0.26	0.25	0.25
NaCl	0.43	0.39	0.39
Calcium monophosphate	0.52	0.43	0.43
Premix 0.5%^1^	0.5	0.5	0.5
Feed additives^2^	0.33	0.33	0
**Chemical composition (g kg**^**−1**^** of experimental diets)**			
Crude protein	170.9	170.2	169.9
Crude fat	37.65	39.05	40.45
Crude fibre	27.14	29.64	30.14
Calcium	7.07	7.00	7.14
Phosphorus	5.47	5.54	5.43
Phytin phosphorus	2.47	2.08	2.04
Iron	0.204	0.209	0.205
Copper	0.015	0.016	0.015
Zinc	0.167	0.169	0.040
Lactic acid	0.885	5.28	5.00

Feeding groups: *C* control group, *FRA* weaners receiving a diet with 8% FRSMb and feed additives, *FR* weaners receiving a diet with 8% FRSMb without feed additives.

^1^PremixMineral-vitamin premix, content in 1 kg: Ca 240 g, K 1 g, Fe 20 g, Mn 11 g, Cu 2.5 g, Se 60.0 mg, I 120 mg, Co 150 mg, vit. A 1,600,000 IU, vit. D3 200,000 IU, vit. E 10.0 g, vit. K 600 mg, vit. B1 500 mg, vit. B2 1400 mg, vit. B6 800 mg, vit. B12 10.0 mg, nicotinic acid 4.0 g, pantothenic acid 4 g, chloric choline 40 g, folic acid 300 mg.

^2^Feed additives content in 1 kg feed: ZnO (78%) 0.160 g Zn; mixture of formic and propionic acid 3:1 (2.5 g); E.coli phytase (0.15 g—5000 FTU/g); xylanase, beta-glucanase, (0.15 g – 12,200 U/g^−1^; 1520 U/g^−1^, respectively); pentosanase, hemicellulase and enzymes which can hydrolyse pectic substances (0.10 g); *Saccharomyces cerevisiae* (0.20 g).

### Chemical analysis

The following were determined in the compound feed and in FRSMb:crude protein, crude fat, and crude fibre^64^. The contents of calcium, zinc, copper and iron were determined by atomic absorption spectrometry, and phosphorus content according to Fiske and Subbarow^65^. The content of phytin phosphorus was determined in the feed and FRSMb according to Oberleas^66^; lactic acid content according to Taylor^67^.

The nutritional value of the diets (except for zinc) was in accordance with nutritional recommendations for pigs^68^. Throughout the experiment the pigs were fed ad libitum and had free access to water.

### Experimental procedures

After the final weighing before slaughter (~35 kg), blood was drawn for analysis from the jugular vein of 6 pigs from each group (one barrow per pen with average body weight for the pen, 18 pigs in total). Then the same animals were slaughtered, and biological material, i.e. the gastrointestinal tract and its contents, was collected for physical and microbiological analysis. Liver samples were also collected from each animal for analysis of redox parameters.

### Laboratory analyses

#### Biochemical and haematological analyses

Immediately after the blood was collected it was divided into portions and placed in test tubes containing heparin as an anticoagulant (biochemical analyses) and EDTA (haematological analyses). Then the samples in heparin tubes were centrifuged for 10 min at 3000 *g*, and the plasma was stored at − 80 °C until analysis. The following were determined in fresh whole blood (EDTA tubes): red blood cell count (RBC), haemoglobin level (Hb), haematocrit (Ht), white blood cell count (WBC) and the percentage composition of white blood cells (leukogram), i.e. the percentage of neutrophils (NEU), lymphocytes (LYM), monocytes (MONO), eosinophils (EOS) and basophils (BAS). Measurements were made using an automatic haematological analyser (Abacuss Junior Vet, Diatron, Hungary).

The content of uric acid (UA), urea (UREA), creatinine (CREAT), and bilirubin (BIL) in the plasma was measured using an automatic biochemical analyser (Plasma Diagnostic Instruments, Horiba, Kyoto, Japan). The concentrations of iron (Fe), copper (Cu), zinc (Zn) and selenium (Se) in the plasma were determined by inductively coupled plasma optical emission spectrometry using the iCAP 6500 duo ICP-OES spectrometer (Thermo Fisher Scientific, Cambridge, UK), equipped with a charge injection device detector.

The titre of class G, A and M immunoglobulins (IgG, IgA, IgM) and IL-6 in the plasma was measured using ELISA immunoenzymatic assays (Fine Test, Wuhan, China) and a Bigenet UMV 340 reader.

The total antioxidant potential of the plasma (FRAP), the level of malondialdehyde (MDA), lipid hydroperoxides (LOOH), and vitamin C, and the activity of superoxide dismutase (SOD) and catalase (CAT) in the blood plasma and liver homogenates were determined according to Czech et al.^69^.

#### Measurements of the gastrointestinal tract

Following evisceration, the gastrointestinal tract was laid out on a table top, where fragments of connective tissue and the mesentery were removed. Then the individual sections of the gastrointestinal tract were straightened out and a measuring tape was used to measure the length of the stomach [(fundus (*fundus ventriculi*) and stomach body (*corpus ventriculi*)], small and large intestine. The measurements were used to determine the total length of the intestines and the total length of the small and large intestine. The pH of the digesta was measured directly using a Testo 206 pH meter.

#### Measurement of the viscosity of the gastrointestinal contents

Viscosity was assessed in the material taken from the duodenal part. Dynamic viscosity was determined using a Zwick/Roell Proline BDO-FB0.5TS testing machine (Zwick GmbH & Co, Ulm, Germany) equipped with a back extrusion system. The measuring components of the system are a beaker (50 mm diameter, 150 mm high) and a dedicated piston with a conical bottom (40 mm diameter, 23.5 mm total height). Prior to the measurements, all samples were conditioned in a water bath to a temperature of 37–38 °C. Viscosity was measured as the difference in the flow force and flow rate of the gastrointestinal contents in the annular gap between the piston and the beaker expressed in η (Pa s). Each measurement was made in 4 cycles (50, 100, 200 and 400 mm/min) using testXpert II software for viscosity analysis.

#### Content of microorganisms in the gastrointestinal tract

An incision was made in the intestine with a sterile scalpel, and the material for microbiological analysis was immediately collected from the intestinal lumen into sterile containers and immediately cooled to 4–8 °C. The samples were transported to the laboratory within 4 hours after collection, where they were homogenized (Unidrive X 1000 CAT, GMBH Germany). Then serial decimal dilutions were made in sterile Ringer’s solution with Tween 80 and incubated on microbiological media according to the standard^70^. The following were determined in each sample: total bacterial count according to PN-EN ISO 4833-2^71^; total number of yeasts according to PN-ISO 21527-1/2^72^; total number of coliform bacteria according to PN-ISO 4832:2007^73^; total number of *Escherichia coli* bacteria according to PN-ISO-16649-2^74^; and total number of *Clostridium perfringens* bacteria according to PN-EN ISO 7937^75^. Each culture on solid substrates was conducted in duplicate. The numbers of microorganisms were expressed as colony forming units (CFU) per gram of the tested material. The result for one animal was expressed as the mean of replicates of the CFU count per g of faeces. The procedure for determination of microbiological parameters is described in detail in Wlazlo et al.^25^.

### Statistical analysis

One-way analysis of variance (ANOVA) was carried out in Statistica ver 10.0 software^76^, (StatSoft Inc., Tulsa, OK, USA). When a treatment effect was observed, Tukey’s post-hoc test was used to determine the differences between treatments. The data were presented as means ± SEM, and a value of P ≤ 0.05 was considered statistically significant.

## Data Availability

The datasets used and/or analysed during the current study available from the corresponding author on reasonable request.
